# Exploring the antimicrobial and antioxidant properties of *Lentzea flaviverrucosa* strain E25-2 isolated from Moroccan forest soil

**DOI:** 10.3389/fmicb.2024.1429035

**Published:** 2024-07-22

**Authors:** Said Rammali, Alin Ciobică, Mohamed El Aalaoui, Abdellatif Rahim, Fatima Zahra Kamal, Khadija Dari, Abdelkrim Khattabi, Laura Romila, Bogdan Novac, Antoneta Petroaie, Bouchaib Bencharki

**Affiliations:** ^1^Laboratory of Agro-Alimentary and Health, Faculty of Sciences and Techniques, Hassan First University of Settat, Settat, Morocco; ^2^Department of Biology, Faculty of Biology, Alexandru Ioan Cuza University of Iasi, Iași, Romania; ^3^Center of Biomedical Research, Romanian Academy, Iasi Branch, Iași, Romania; ^4^Academy of Romanian Scientists, Bucharest, Romania; ^5^CENEMED Platform for Interdisciplinary Research, “Grigore T. Popa” University of Medicine and Pharmacy of Iasi, Iasi, Romania; ^6^Regional Center of Agronomic Research of Settat, Settat, Morocco; ^7^Laboratory of Biochemistry, Neurosciences, Natural Resources and Environment, Faculty of Sciences and Techniques, Hassan First University of Settat, Settat, Morocco; ^8^Higher Institute of Nursing Professions and Health Technical (ISPITS), Marrakech, Morocco; ^9^Laboratory of Physical Chemistry of Processes and Materials, Faculty of Sciences and Techniques, Hassan First University, Settat, Morocco; ^10^Department of Chemistry, “Ioan Haulica” Institute, Apollonia University, Iași, Romania; ^11^Urology Department, Grigore T. Popa University of Medicine and Pharmacy, Iași, Romania; ^12^Family Medicine Department, Grigore T. Popa University of Medicine and Pharmacy, Iași, Romania

**Keywords:** *Lentzea flaviverrucosa*, antimicrobial activity, antioxidant activity, 16S rRNA gene sequences, hemolytic activity, GC-MS

## Abstract

The alarming rise in antimicrobial resistance (AMR) has created a significant public health challenge, necessitating the discovery of new therapeutic agents to combat infectious diseases and oxidative stress-related disorders. The *Lentzea flaviverrucosa* strain E25-2, isolated from Moroccan forest soil, represents a potential avenue for such research. This study aimed to identify the isolate E25-2, obtained from soil in a cold Moroccan ecosystem, and further investigate its antimicrobial and antioxidant activities. Phylogenetic analysis based on 16S rRNA gene sequences revealed the strain’s classification within the *Lentzea* genus, with a sequence closely resembling that of *Lentzea flaviverrucosa* AS4.0578 (96.10% similarity). Antimicrobial activity in solid media showed moderate to strong activity against *Staphylococcus aureus* ATCC 25923, *Bacillus cereus* strain ATCC 14579, *Escherichia coli* strain ATCC 25922, *Candida albicans* strain ATCC 60193 and 4 phytopathogenic fungi. In addition, ethyl acetate extract of this isolate demonstrated potent antimicrobial activity against 7 clinically multi-drug resistant bacteria. Furthermore, it demonstrated antioxidant activity against 2,2-diphenyl-1-picrylhydrazyl (DPPH) and 2,2′-azino-bis (3-ethylbenzothiazoline-6-sulfonic acid) (ABTS) free radicals, as well as a significant increase in ferric reducing antioxidant power. A significant positive correlation was observed between antioxidant activities and total content of phenolic compounds (*p* < 0.0001), along with flavonoids (*p* < 0.0001). Furthermore, gas chromatography-mass spectrometry (GC-MS) analysis revealed the presence of amines, hydroxyl groups, pyridopyrazinone rings, esters and pyrrolopyrazines. The *Lentzea* genus could offer promising prospects in the fight against antibiotic resistance and in the prevention against oxidative stress related diseases.

## 1 Introduction

The advent of antibiotics in the 20th century revolutionized the treatment of infectious diseases, marking a new era in medical science (Browne et al., 2020). These “magic bullets” have saved countless lives by effectively targeting and eliminating bacterial pathogens (da Cunha et al., 2019). However, the widespread and often indiscriminate use of antibiotics has created a significant public health challenge with antimicrobial resistance (AMR). Nowadays, antimicrobial-resistant infections have become the third leading cause of death, following cardiovascular diseases and cancer (Meštrović et al., 2023). AMR occurs when microorganisms such as bacteria, viruses, fungi, and parasites evolve mechanisms to withstand the drugs designed to kill them (World Health Organization, 2023). This phenomenon not only renders standard treatments ineffective but also increases the risk of severe illness and death (Tang et al., 2023). In 2019, approximately 1.27 million deaths were attributed to infections caused by antimicrobial resistance (AMR), and nearly 5 million deaths were linked to multidrug-resistant (MDR) infections (Salam et al., 2023). By 2050, this number is expected to rise to 10 million annually, greatly exceeding the mortality rates of cancer (Will 10 Million People Die a Year due to Antimicrobial Resistance by 2050?). One of the earliest known “superbugs,” methicillin-resistant *Staphylococcus aureus* (MRSA), is linked to a high death toll from AMR infections globally (World Health Organization, 2023). Presently, 3.5% of active tuberculosis cases and 18% of previously treated cases are categorized as multidrug-resistant tuberculosis (MDR-TB) worldwide, with increasing concern over extensively drug-resistant tuberculosis (XDR-TB) in many MDR-TB cases (Uddin et al., 2021).

Given the alarming rise in infectious diseases and bacterial resistance, it is essential to discover new natural bioactive compounds as alternatives to antibiotics to combat the progression toward multidrug resistance (MDR). In this context, it was reported that the most effective source for discovering powerful new bioactive compounds is natural products derived from Microorganisms including bacteria (Hug et al., 2020). Bacteria produce secondary metabolites, which are not necessary for survival but provide advantages under specific conditions (Hug et al., 2020). These secondary metabolites exhibit diverse biological functions and are used as antifungals, anticancer, immunosuppressive, and antibiotics compounds (Barka et al., 2016; Jones and Elliot, 2017). *Actinobacteria* are massive antibiotic producers, accounting for almost two-thirds of all known antibiotics used in medicine (Lu et al., 2019). They are an important and inexhaustible source for the natural production of antibiotics, and they remain a source of interest for the discovery of new antibiotics (Genilloud, 2017). Furthermore, the *Actinobacteria* are recognized for their biocontrol potential due to their ability to produce bioactive compounds (Barka et al., 2016).

The *Lentzea* genus is a Gram-positive group, aerobic, non-motile, filamentous *Actinobacteria* characterized by branching aerial mycelia that fragment into rod-like elements (Cao et al., 2015). The *Lentzea* genome contains between 68.6 and 79.6% guanine and cytosine (G + C) (Cao et al., 2015). As part of our program focused on isolating *Actinobacteria* from the soils of the Azrou forest in the Fez-Meknes region of Morocco, aiming to explore their chemical diversity and potential in antibiotic discovery (Rammali et al., 2022), we came across a rare *Actinobacteria* strain identified as *Lentzea* sp. strain E25-2. Remarkably, the bioactive compounds produced by this genus, except for *Lentzea violacea*, have not been widely documented (Hussain et al., 2017). In this study, we first identified the genus of isolate E25-2, obtained from the soil of a cold Moroccan ecosystem. Secondly, our aim is to explore its biological properties, particularly focusing on its antimicrobial and antioxidant activities.

## 2 Materials and methods

### 2.1 Soil sampling, isolation and conservation

In February and early March 2019, a pure strain, designated E25-2, was isolated from a soil sample from an unexploited cold ecosystem in the Azrou forest, in the Fez-Meknes region of Morocco (GPS: 33° 26′ 28″ N 5° 13′ 22″ W). The soil sample was collected at five separate points over an area of 400 m^2^ following the described sampling method by Sengupta et al. (2015) and underwent pretreatment by the enrichment method (Kitouni et al., 2005). For *Actinobacteria* isolation, M2 medium supplemented with 50 mg/L actidione (cycloheximide) (Bouaziz et al., 2016) was used. Strain E25-2 was grown on ISP2 agar medium, incubated at 28°C, and subsequently stored temporarily in inclined tubes at 4°C and long-term in a 20% glycerol solution at −20°C (Marimuthu et al., 2020).

### 2.2 Genotypic identification

The genomic DNA extraction from isolate E25-2, the amplification and sequencing reactions, was carried out following the protocol described in our previous work (Rammali et al., 2022, 2024). The obtained 16S rRNA gene sequence for isolate E25-2 was aligned with related *Lentzea* genus sequences using MEGA-X software (Kumar et al., 2018). Phylogenetic tree construction employed the neighbor-joining tree method (Saitou and Nei, 1987). Sequence similarity was assessed using GenBank and EzBioCloud genomic databases.

### 2.3 Phenotypic characteristics of E25-2 isolate

According to Shirling and Gottlieb (1966), the cultural characteristics of isolate E25-2 were observed on ISP (International *Streptomyces* Project) media (ISP-1, ISP-2, ISP-4, ISP-5 and GYEA). The cell morphology of the isolate was determined by light microscopy with a digital camera (Olympus CX43RF) in its native condition and subsequent to Gram staining, employing the slide culture method (Williams and Cross, 1971). Melanoid pigment production, tolerance to various concentrations of NaCl (1, 2, 3, 4, 5, 7 and 10%), tolerance to different pH values (4.63, 5.33, 6.41, 7.31, 8.28, 9.27 and 10.03), growth at different temperature levels (4, 28, 37 and 46°C) and assimilation of carbohydrates and their derivatives as the sole carbon source were evaluated in this study (Singh et al., 2016).

### 2.4 Preliminary evaluation of antimicrobial activity

Primary screening to evaluate the antimicrobial activity of the E25-2 isolate was performed using the double-layer method described by Badji et al. (2005), utilizing four agar media (Bennett, ISP1, ISP2, and GYEA) against various microorganisms: *Escherichia coli* ATCC 25922, *Staphylococcus aureus* ATCC 25923, *Bacillus cereus* ATCC 14579, and *Candida albicans* ATCC 60193 (obtained from the Institut Pasteur collection in Casablanca, Morocco), as well as *Fusarium* sp. MN944575, *Fusarium* sp. MN944576, and *Trichoderma longibrachiatum* M37 (these phytopathogenic fungi were obtained from the Laboratory of Agro-Alimentary and Health, Faculty of Sciences and Techniques, Hassan First University of Settat, Morocco).

### 2.5 Secondary evaluation of antimicrobial activity

#### 2.5.1 Preparation of the extract of E25-2 isolate

The fermentation and secondary metabolite extraction of the E25-2 strain were conducted according to the protocol outlined in our previous work (Rammali et al., 2022, 2023, 2024). Antimicrobial activity in organic extracts from isolate E25-2.

The assessment of the antimicrobial efficacy of extracts derived from the organic phase of the E25-2 isolate was conducted using the disk diffusion method (Badji et al., 2005). Evaluation involved testing against *Escherichia coli* strain ATCC 25922, *Staphylococcus aureus* strain ATCC 25923, *Bacillus cereus* ATCC 14579, *Candida albicans* ATCC 60193, and 5 clinical MDR strains (*Enterococcus* strain 18k1386, *Staphylococcus aureus* strain 18k1052, *Proteus vulgaris* strain 16C1737, *Neisseria gonorrhoeae* strain 16D1170, *Escherichia coli* strain 16D1150). The antibiotic resistance profile of the clinical MDR strains tested was verified against 16 antibiotics (Supplementary Table S3). These MDR strains were obtained from the collection of the Pasteur Settat Morocco Medical Analysis Laboratory. Prior to antimicrobial testing, bacterial cells were harvested and adjusted to an optical density (OD) of 0.08–0.13 at 625 nm, approximately corresponding to 10^6^ CFU/mL, using a spectrophotometer (Selectra VR2000, Barcelona, Spain) (CASFM, 2020). Similarly, for antifungal activity assessments, inoculum optical densities were maintained within the 0.18–0.20 range at 623 nm, corresponding to a concentration of approximately 10^6^ spores/mL (Bastide et al., 1986). DMSO-impregnated discs of equivalent volume served as negative controls. Streptomycin was used for antibacterial activity assessment, while cycloheximide served as the positive control for antifungal activities.

### 2.6 Total phenolic and flavonoid contents

The total phenolic contents quantification in the ethyl acetate extract of the E25-2 isolate was performed using the Folin–Ciocalteu method, as outlined by Bensadón et al. (2010). The absorbance was recorded at 760 nm, with gallic acid used as standard. Additionally, the total flavonoid contents were determined according to Bahorun et al. (2006). The absorbance was recorded at 415 nm, quercetin as standard.

### 2.7 *In vitro* antioxidant activity tests

The DPPH assay of ethyl acetate extract of E25-2 isolate was carried out following the protocol of Blois (1965). The absorbance was measured at 517 nm. Ascorbic acid was used as a positive antioxidant. The ABTS assay of ethyl acetate extract of E25-2 isolate was assessed following the method previously adopted by Re et al. (1999). The result of the reaction was recorded by measuring absorbance at 734 nm. Trolox was used as a positive antioxidant. Concerning FRAP, it was determined according to Oyaizu (1986). The absorbance was measured at 700 nm. With ascorbic acid used as the positive antioxidant. In all assays, a spectrophotometer (Selectra VR2000, Barcelona, Spain) was employed to measure the absorbance.

### 2.8 Evaluating toxicity of ethyl acetate extract of E25-2 isolate

The UV–visible spectrophotometer was used to measure the absorption spectra of the ethyl acetate extract from the E25-2 isolate, solubilized in DMSO, across the wavelength range of 190 to 850 nm (HACH lange DR6000) (Bastide et al., 1986).

### 2.9 Evaluating toxicity of E25-2 isolate using *in vitro* method hemolysis

The hemolytic activity of ethyl acetate extract of E25-2 isolate was performed following the methods described by our group (Rammali et al., 2024). The degree of hemolysis was expressed as the hemolysis rate, calculated according to the following formula:


$$\mathrm{Hemolysis rate}=\left[\frac{\left(\begin{array}{l} OD\;\left(\mathrm{Test}\right)-\\ OD\;\left(\mathrm{Negative control}\right) \end{array}\right)}{\begin{array}{l} OD\;\left(\mathrm{Positive control}\right)-\\ OD\;\left(\mathrm{Negative control}\right) \end{array}}\;\right]\times 100\%$$

### 2.10 Gas chromatography-mass spectrometry analysis

The gas chromatography-mass spectrometry (GC-MS) analysis was performed according to the protocol detailed in our previous work (Rammali et al., 2022, 2024).

### 2.11 Statistical analysis

The experiments were conducted in triplicate, and the results were presented as the mean value along with the standard deviation (SD). To assess differences between groups in phenolic compound, flavonoid compound, and antioxidant activity assays, the GraphPad Prism 8.4.3 software was used. Specifically, we performed a standard two-way ANOVA followed by Tukey’s multiple comparisons test. Statistical significance was set at *p* < 0.05. Pearson correlation analysis was conducted using GraphPad Prism 8.4.3 software to assess the relationship between total phenolic and flavonoid compounds and antioxidant activity.

## 3 Results

### 3.1 16S rRNA PCR and phylogenetic approaches

**Figure 1:**
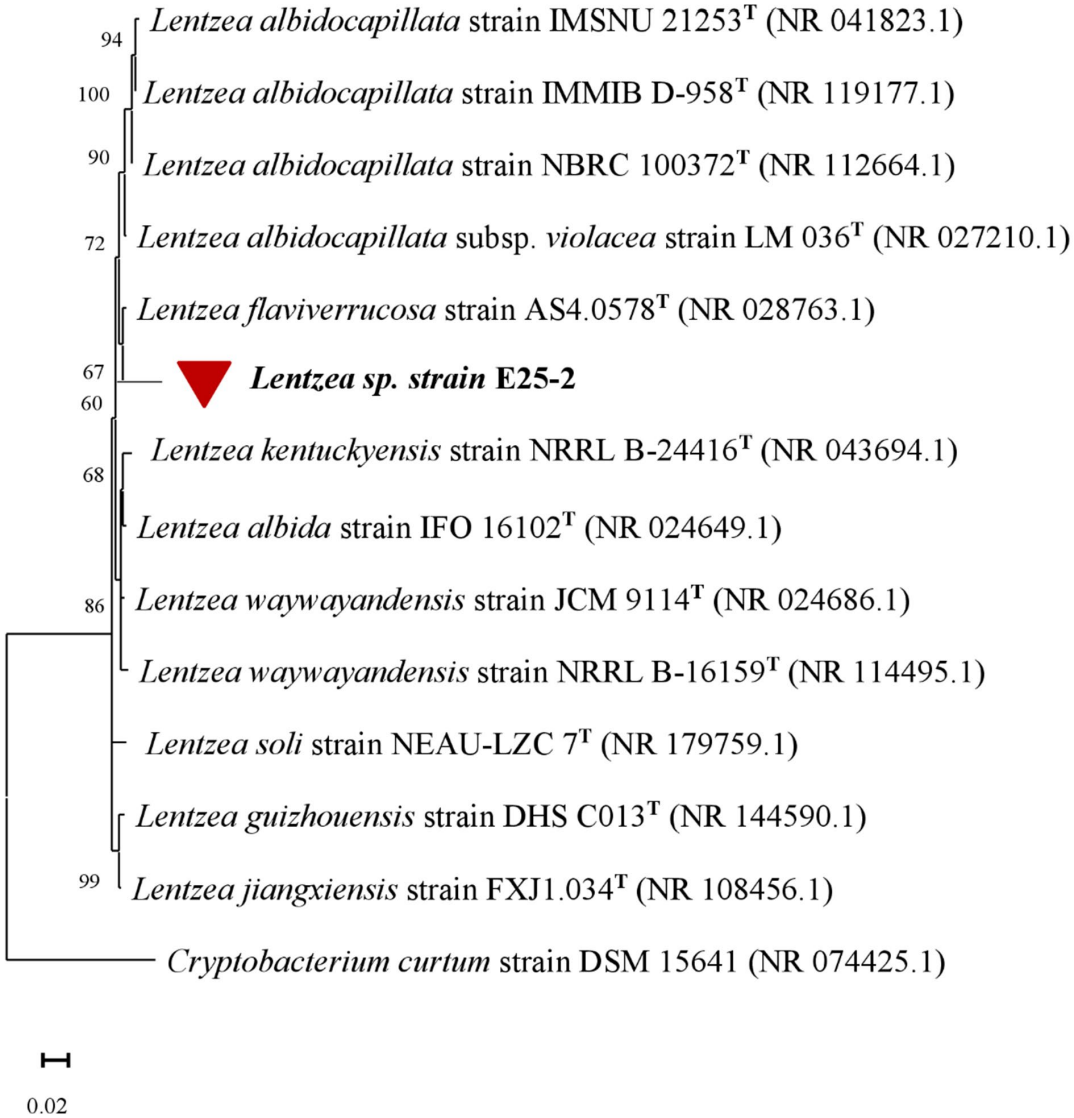
The phylogenetic tree, based on the 16S rRNA gene, was constructed using the Neighbour-Joining Tree statistical method using MEGA X software. The 16S rRNA gene sequence analysis illustrates the evolutionary relationship between *Lentzea* sp. strain E25-2, indicated by a red circle, and its closest known taxa. The bar (0.02) represents the number of substitutions per nucleotide position. GenBank accession numbers are provided in brackets. *Cryptobacterium curtum* was used as the outgroup in the analysis. T, type strain.

The sequencing analysis revealed that the almost complete sequence of the 16S rRNA gene of isolate E25-2 was 1,103 bp (GenBank accession number: OR865862) (Supplementary Table S1). By comparing the aligned sequence of the 16S rRNA gene of isolate E25-2 with the corresponding partial sequences of the 16S rRNA gene from type strains accessible in the GenBank genomic database, we were able to determine the taxonomic classification of isolate E25-2. According to the results, strain E25-2 demonstrates approximately 97% similarity with the genus *Lentzea* (Supplementary Table S1). The phylogenetic tree indicated that the E25-2 strain exhibited a 16S rRNA sequence similarity of 96.10% with *Lentzea flaviverrucosa* AS4.0578 (Figure 1).

### 3.2 Phenotypic analysis of *Lentzea* sp. E25-2 isolate

The culture characteristics analysis revealed that isolate E25-2 exhibits growth on all tested agar culture media (ISP1, ISP2, ISP4, ISP5, ISP7, and GYEA) (Table 1). Colonies of isolate E25-2 are star-shaped, ranging from 1 to 6 mm in diameter (Supplementary Figure S1). The aerial mycelium, observed on the surface of the medium, has a whitish, non-powdery appearance, while the substrate mycelium on the underside of the medium, has an orange-yellow color (Supplementary Table S2). Under light microscopy in the fresh state and after Gram staining, isolate E25-2 was identified as a filamentous, immobile Gram-positive bacterium. It displayed segmented, branched aerial mycelium under observation (Supplementary Figure S2). The isolate exhibits robust growth on all tested agar types (ISP1, ISP2, ISP4, ISP5, ISP7 and GYEA) after 1 week of aerobic incubation at 28°C. The isolate E25-2 is negative for melanoid pigment production. In fact, no diffusible pigments were observed on the culture media. Moreover, it grows in a pH range from 5.33 to 10.03, with optimal growth at pH 8.28, and is unable to grow at pH below 5 (Table 1). The isolate E25-2 tolerates NaCl concentrations from 1 to 6%, but no growth above 7%. The carbon source identification test showed that this isolate can assimilate all carbohydrate compounds: D-xylose, mannitol, D-raffinose, cellobiose, sucrose, D-galactose, melibiose, ribose, D-mannose, D-fructose, trehalose, maltose, and glucose except Melezitose and D-arabinose (Table 1).

**Table 1:** Characteristics of isolate E25-2.

Characteristics	E25-2
**Assimilation**	
Ribose	+
Melezitose	−
D-arabinose	−
Mannitol	3+
Trehalose	3+
Cellobiose	3+
Sucrose	3+
Raffinose	2-
Xylose	2+
Melibiose	2+
Mannose	3+
Fructose	3+
Galactose	3+
Maltose	2+
Glucose	+
**Growth on**	
ISP1	3+
ISP2	3+
ISP4	3+
ISP5	3+
ISP7	3+
GYEA	3+
**pH tolerance**	
4.63	−
5.33	+
6.41	+
7.31	3+
8.28	3+
9.27	3+
10.03	3+
**NaCl tolerance**	
1%	3+
2%	3+
3%	3+
4%	3+
5%	2+
6%	+
7%	−
10%	−
**Growth on**	
4°C	−
28°C	3+
37°C	2+
46°C	−

−, no growth; +, low growth; 2+, intermediate growth; 3+, good growth.

### 3.3 Antimicrobial potential of isolate E25-2

**Figure 2:**
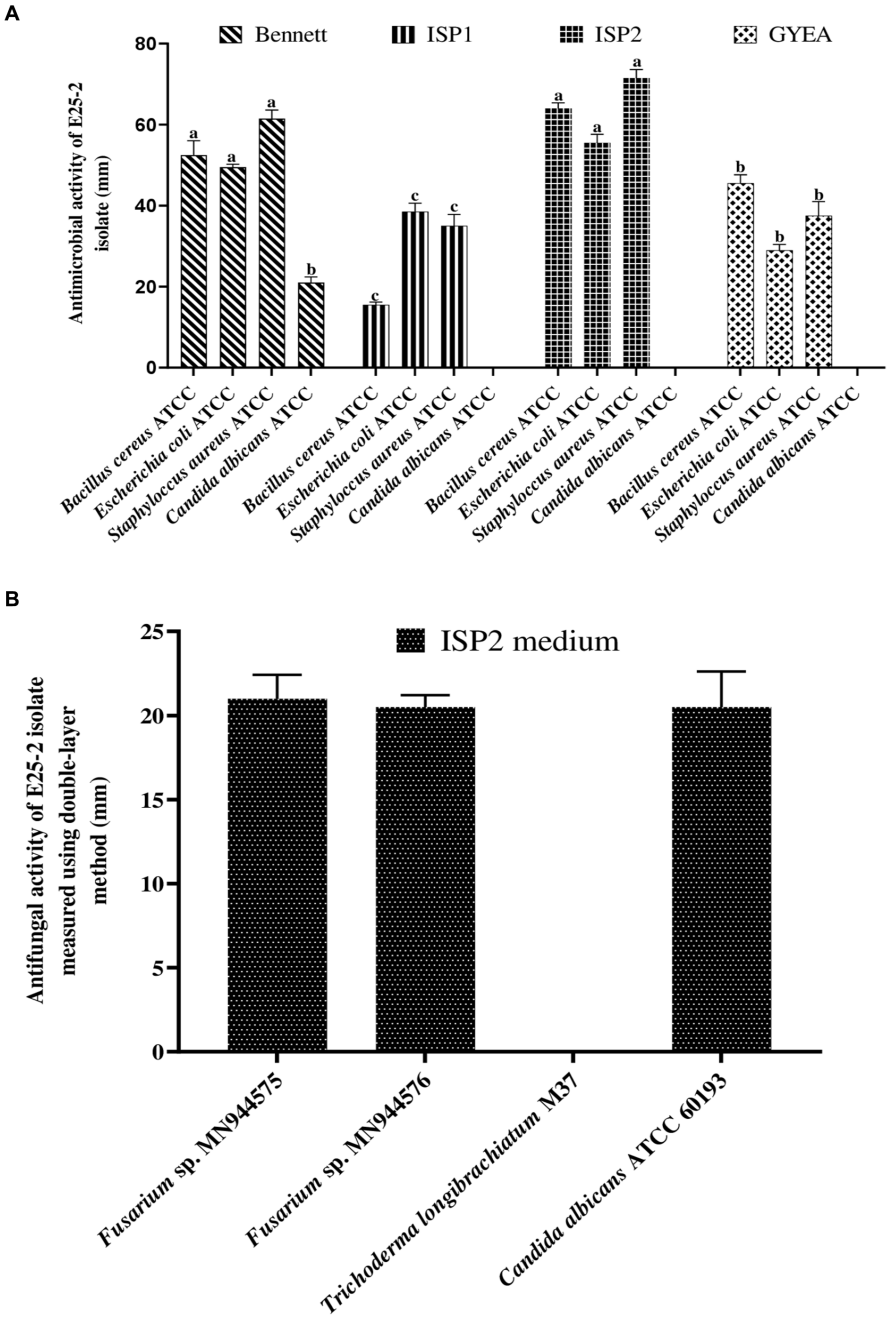
Antibacterial **(A)** and antifungal **(B)** activities of isolate E25-2 assessed via double layer method on solid media.

**Figure 3:**
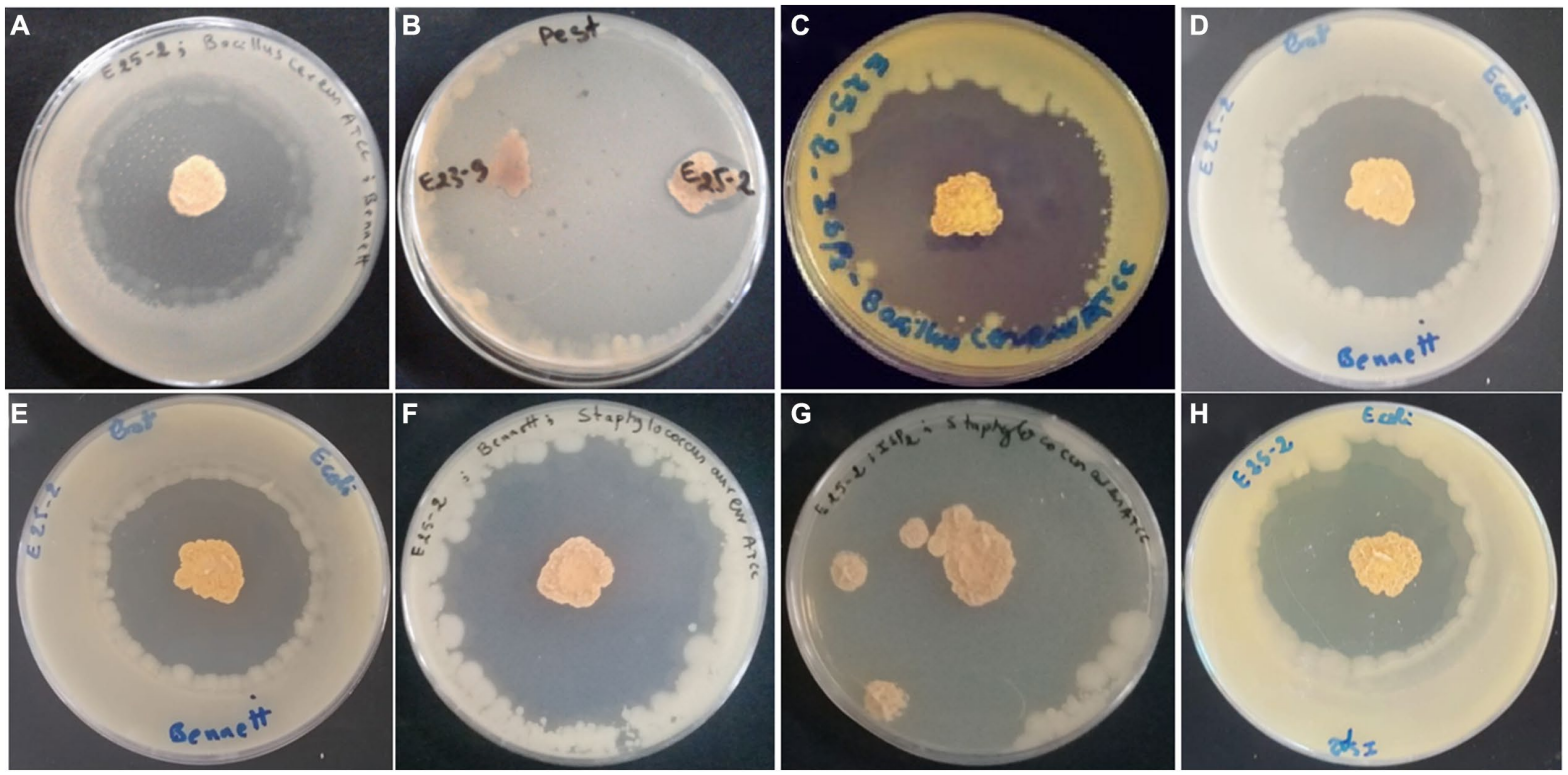
Antimicrobial activity of *Lentzea* sp. isolate E25-2 using the double-layer method on ISP1, ISP2, Bennet and GYEA media. **(A)** Antibacterial activity of E25-2 against *Bacillus cereus* ATCC 14579 using Bennett medium. **(B)** Antifungal activity of E25-2 against *Fusarium* sp. strain MN944575. **(C)** Antibacterial activity of E25-2 against *Bacillus cereus* ATCC 14579 using ISP2 medium. **(D,E)** Antibacterial activity of E25-2 against *Escherichia coli* ATCC 25922 using Bennett medium. **(F)** Antibacterial activity of E25-2 against *Staphylococcus aureus* ATCC 25923 using Bennett medium. **(G)** Antibacterial activity of E25-2 against *Staphylococcus aureus* ATCC 25923 using ISP2 medium. **(H)** Antibacterial activity of E25-2 against *Escherichia coli* ATCC 25922 using ISP2 medium.

Isolate E25-2 exhibited antimicrobial activity on all the agar media tested, with significantly higher activity observed on ISP2 and Bennett media. Conversely, ISP1 and GYEA provided less favorable conditions for secondary metabolite production (Figures 2, 3). The antimicrobial assays revealed significant activity of isolate E25-2 against both Gram-positive bacteria (*Staphylococcus aureus* ATCC 25923, *Bacillus cereus* ATCC 14579) and Gram-negative bacteria (*Escherichia coli* ATCC 25922), as well as *Candida albicans* ATCC 60193. Furthermore, isolate E25-2 demonstrated notable antimicrobial activity against two phytopathogenic fungi *Fusarium* sp. MN944576 and *Fusarium* sp. MN944575, as well as *Trichoderma longibrachiatum* (M37), which is known to cause invasive lung infections.

**Figure 4:**
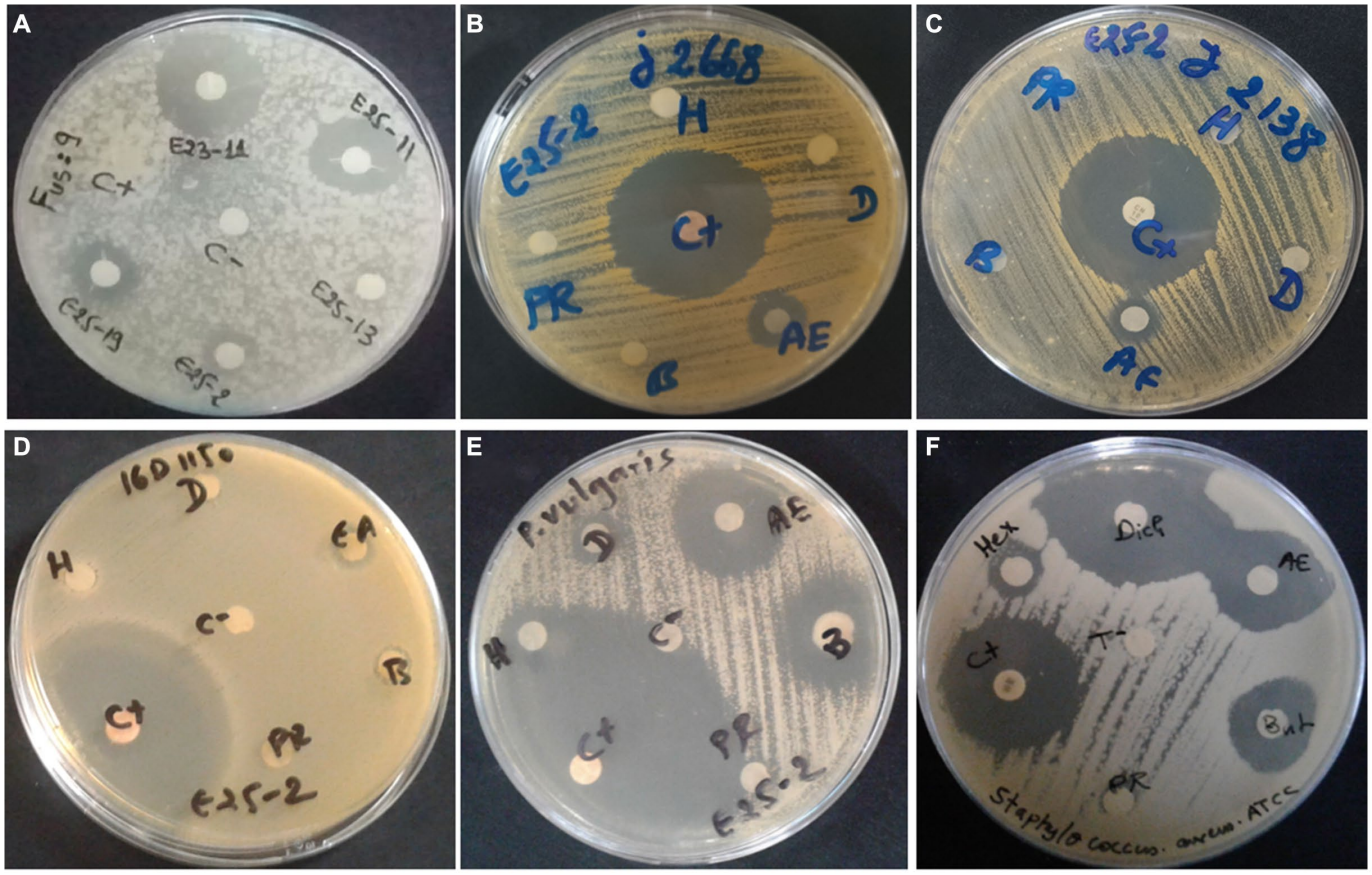
Antimicrobial activity of *Lentzea* sp. isolate E25-2 against MDR clinical bacteria and plant pathogenic fungi using disk diffusion method. C (+), Streptomycin (positive control); C (−), negative control; H, hexane; D, dichloromethane; EA, ethyl acetate; B, butanol; PR, residual phase. **(A)** Antifungal activity of E25-2 and other isolates against *Fusarium* sp. strain MN944575. **(B)** Antibacterial activity of E25-2 against clinical *Staphylococcus aureus* 23J2668. **(C)** Antibacterial activity of E25-2 against clinical *Staphylococcus saprophyticus* 23J2138. **(D)** Antibacterial activity of E25-2 against clinical *Escherichia coli* 16D1150. **(E)** Antibacterial activity of E25-2 against clinical *Proteus vulgaris* 16C1737. **(F)** Antibacterial activity of E25-2 against *Staphylococcus aureus* ATCC 25923.

Antimicrobial activity, assessed using n-hexane, dichloromethane, ethyl acetate and n-butanol, revealed that ethyl acetate was the most effective solvent for extracting the main antimicrobial agents (with zones of inhibition ranging between 6.5 ± 0.71 and 25 ± 1.41 mm). In addition, isolate E25-2 underwent specific secondary screening, involving MDR pathogenic bacteria and phytopathogenic fungi (Figure 4; Table 2; Supplementary Table S3). Both primary and secondary screening results indicated a more pronounced activity of this isolate against Gram-positive bacteria, compared to Gram-negative bacteria.

**Table 2:** Antimicrobial activity of E25-2 isolate using disk diffusion method according to extraction solvents.

Target strains	Antimicrobial activity of E25-2 isolate (mm)				
	CP^A^	Hex^B^	Dich^B^	EA^B^	But^B^
*Staphylococcus aureus* ATCC 25923	27.51 ± 2.12	—	—	10.50 ± 0.71	—
*Bacillus cereus* ATCC 14579	28.00 ± 4.24	—	—	11.50 ± 0.71	—
Clinical *Enterococcus* 18k1386	11.00 ± 1.41	—	—	10.50 ± 0.71	—
Clinical *Staphylococcus aureus* 18k1052	24.00 ± 1.41	—	—	11.00 ± 050	—
Clinical *Proteus vulgaris* 16C1737	35.50 ± 0.71	11.00 ± 1.41	12.50 ± 0.71	25.00 ± 1.41	23.50 ± 2.12
*Escherichia coli* ATCC 25922	28.00 ± 1.41	—	—	13.50 ± 0.71	—
Clinical *Neisseria gonorrhoeae* 16D1170	25.50 ± 2.12	—	—	06.50 ± 0.71	—
Clinical *Escherichia coli* 16D1150	27.00 ± 1.41	12.50 ± 0.71	9.50 ± 0.71	10.50 ± 0.71	8.50 ± 0.71
*Candida albicans* ATCC 60193	27.00 ± 2,83	—	—	19.00 ± 0.71	—
Clinical *Candida albicans* 23I2445	29.00 ± 1.00	—	—	10.50 ± 0.50	—
Clinical *Staphylococcus aureus* 23J2668	31.00 ± 1.00	—	—	14.50 ± 0.50	—
Clinical *Staphylococcus saprophyticus* 23J2138	30.50 ± 0.50	—	—	11.50 ± 0.50	—

Values expressed are means ± SD (*n* = 2). Columns marked with different letters A and B are significantly different (ordinary one-way ANOVA: Tukey’s multiple comparisons test, *p* < 0.05). CP, positive control (streptomycin); Hex, hexane; Dich, dichloromethane; EA, ethyl acetate; But; butanol; —, no inhibition zone.

### 3.4 The phenolic and flavonoid content of ethyl acetate extract from E25-2 isolate

The findings of the flavonoid and total phenolic contents of the ethyl acetate extract of isolate E25-2 are presented in Table 3. Pearson’s statistical analysis revealed a positive correlation between the concentrations of ethyl acetate extract and total phenolic content (*r* = 0.997; *p* < 0.001), as well as a positive correlation with flavonoid content (*r* = 0.834; *p* < 0.05). These correlations were calculated as the mean of three repetitive measurements.

**Table 3:** Flavonoid and total phenolic contents of ethyl acetate extract of *Lentzea* sp. E25-2 strain.

Concentration of E25-2 ethyl acetate extract (mg/mL)	Total phenols contents (mg GAE/mg extract)	Total flavonoids contents (mg QE/mg extract)
0.10	0.17 ± 0.01	ND
0.20	0.19 ± 0.01	ND
0.30	0.22 ± 0.01	ND
0.40	0.27 ± 0.01	ND
0.50	0.31 ± 0.01	ND
0.60	0.34 ± 0.01	ND
0.70	0.37 ± 0.01	0.02 ± 0.02
0.80	0.40 ± 0.01	0.06 ± 0.02
0.90	0.44 ± 0.01	0.09 ± 0.01
1.00	0.50 ± 0.01	0.14 ± 0.02

ND, not determined; GAE, gallic acid equivalent; QE, quercetin equivalent.

### 3.5 *In-vitro* antioxidant potential of E25-2 isolate

**Figure 5:**
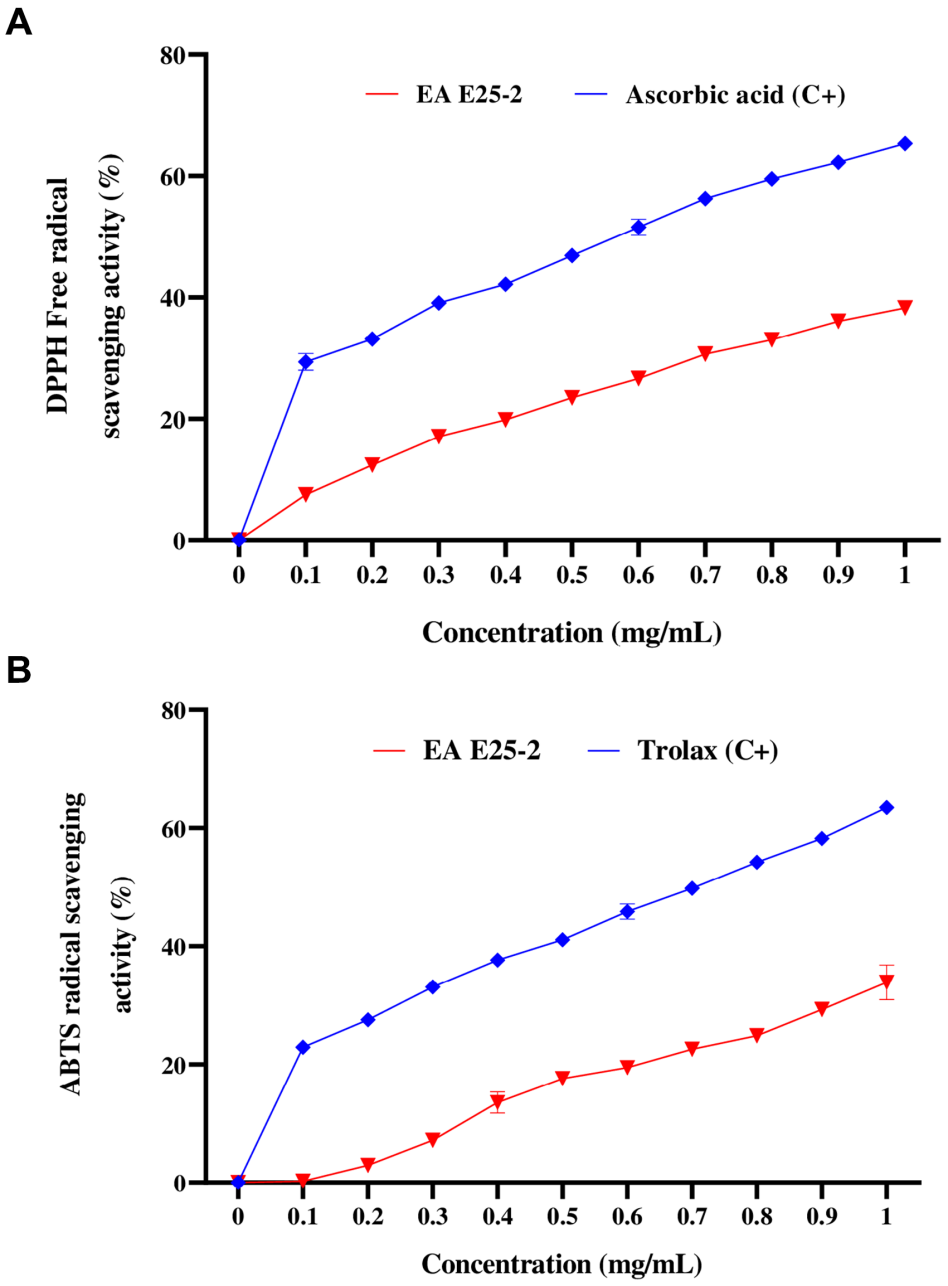
Antioxidant activity of ethyl acetate extract of *Lentzea* sp. E25-2 strain in various antioxidant assays. **(A)** antioxidant activity by DPPH assay and **(B)** antioxidant activity by ABTS assay. Values expressed are means ± SD (*n* = 3). Symbol (****) indicates *p* < 0.0001 significant difference between ethyl acetate extract of *Lentzea* sp. E25-2 isolate and controls. (C+), positive control; (EA), ethyl acetate.

The ethyl acetate extract of isolate E25-2 exhibited lower DPPH and ABTS activities com-pared to the standards (ascorbic acid and Trolox) (*p* < 0.0001) (Figures 5A,B). The results presented in the Figure 5A indicate that the ethyl acetate extract from the E25-2 isolate exhibited significant DPPH free radical scavenging activity between the different tested concentrations (*p* < 0.0001), with inhibition percentages ranging from 2.91 ± 0.92 to 37.78 ± 1.93% for concentrations between 0.2 and 1 mg/mL. In addition, the extract demonstrated high ABTS radical scavenging activity between the different tested concentrations (*p* < 0.0001), with inhibition percentages ranging from 1.32 ± 1.96 to 30.58 ± 1.78% over the same concentration range (Figure 5B).

**Figure 6:**
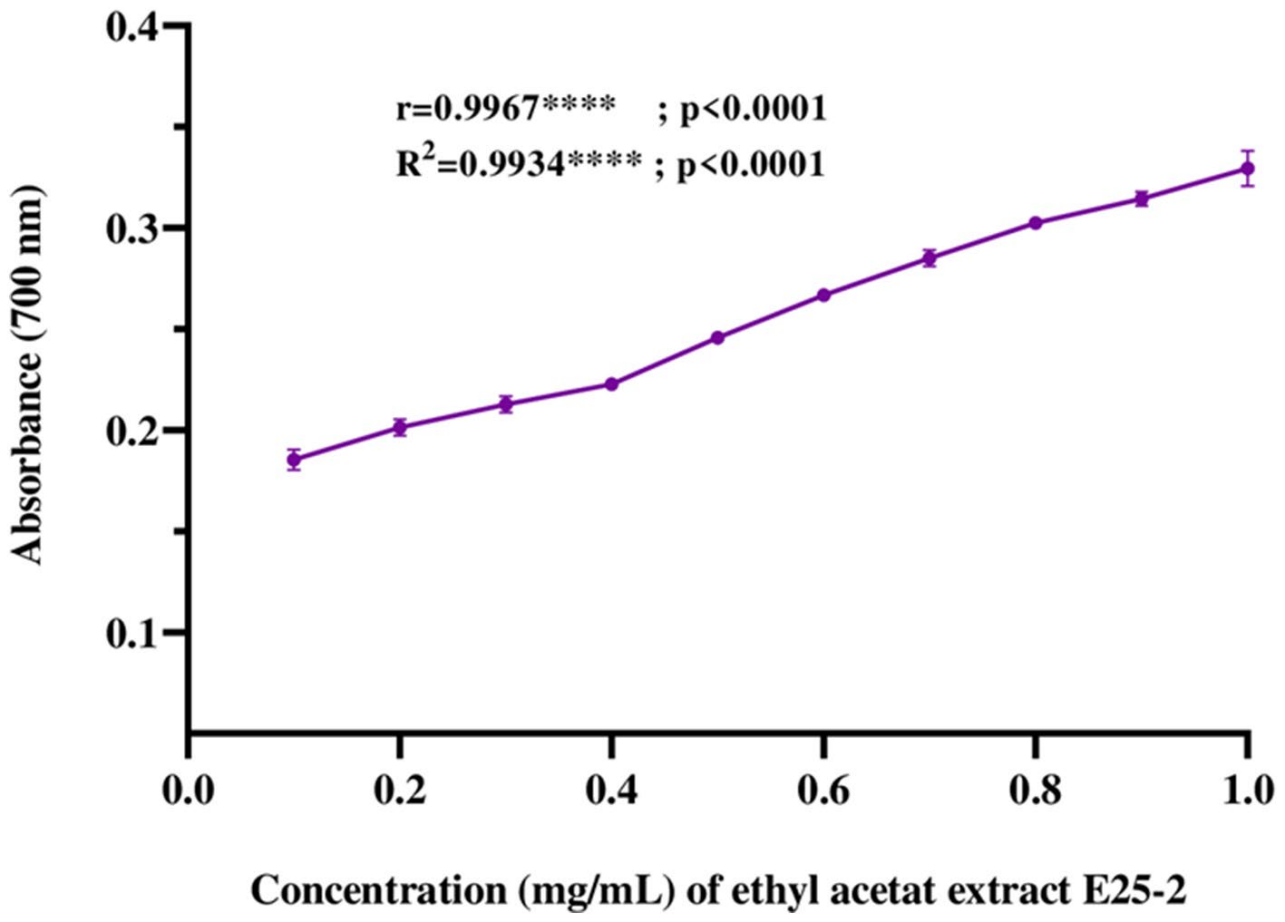
Ferric reducing antioxidant power (FRAP) of ethyl acetate extract of *Lentzea* sp. E25-2: standard deviation analysis for 10 doses (0.1 mg–1 mg) in triplicate (*n* = 3).

Regarding iron antioxidant reducing power (FRAP), the results revealed a significant iron-reducing activity for ethyl acetate extract across the tested concentrations (*p* < 0.0001). This activity showed values from 0.43 ± 0.01 to 0.89 ± 0.02 mg AAE (ascorbic acid equivalent) per milligram of extract (Figure 6).

### 3.6 Correlation between antioxidant activity and total phenol and flavonoid contents

**Figure 7:**
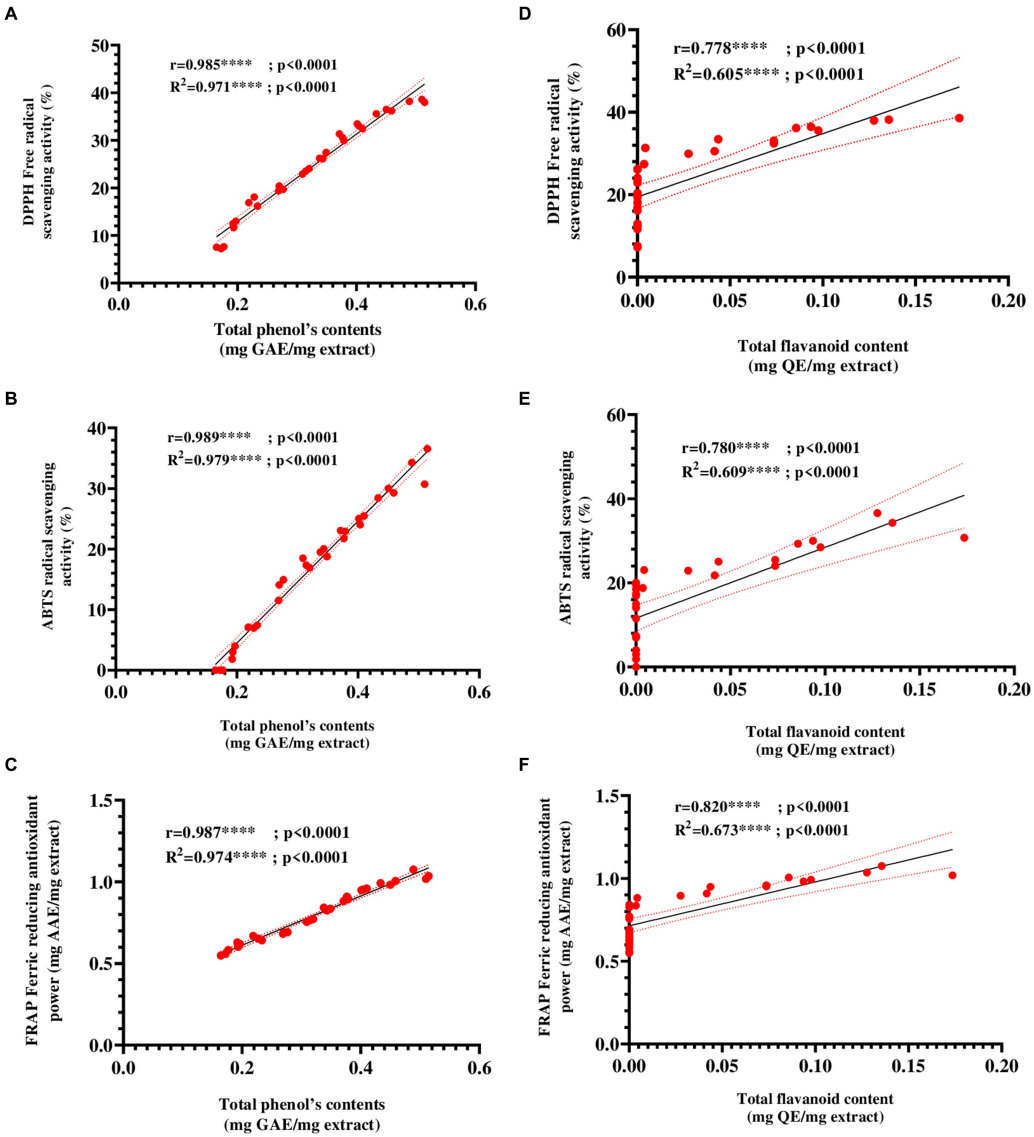
Pearson’s correlation coefficients between total phenolic and flavonoid contents and antioxidant activities ethyl acetate extract of Lentzea sp. E25-2 isolate. **(A)** Pearson correlation between DPPH and total phenolic content. **(B)** Pearson correlation between ABTS and total phenolic content. **(C)** Pearson correlation between FRAP and total phenolic content. **(D)** Pearson correlation between DPPH and flavonoids content. **(E)** Pearson correlation between ABTS and flavonoids content. **(F)** Pearson correlation between FRAP and flavonoids content. Symbol (****) indicates *p* < 0.0001 highly significant between tests.

To evaluate the relationship between the antioxidant capacity of E25-2 ethyl acetate extract and its total phenol and flavonoid contents, a correlation analysis was also carried out. Figures 7A–F presents the Pearson correlation coefficients between the variables. The findings demonstrated a remarkably strong positive association between antioxidant activity, as measured by DPPH, ABTS and FRAP assays, and the polyphenol and flavonoid contents of ethyl acetate extract of *Lentzea* sp. isolate E25-2. A significantly positive correlation was observed between the total polyphenol content (TPC) and the ferric reducing antioxidant power (FRAP) of the E25-2 extract (*r* = 0.987, *p* < 0.0001), as well as between the total flavonoid content (TFC) and FRAP of this extract (*r* = 0.821, *p* < 0.05).

### 3.7 Evaluating toxicity of ethyl acetate extract from isolate E25-2 using UV-visible spectral assay

**Figure 8:**
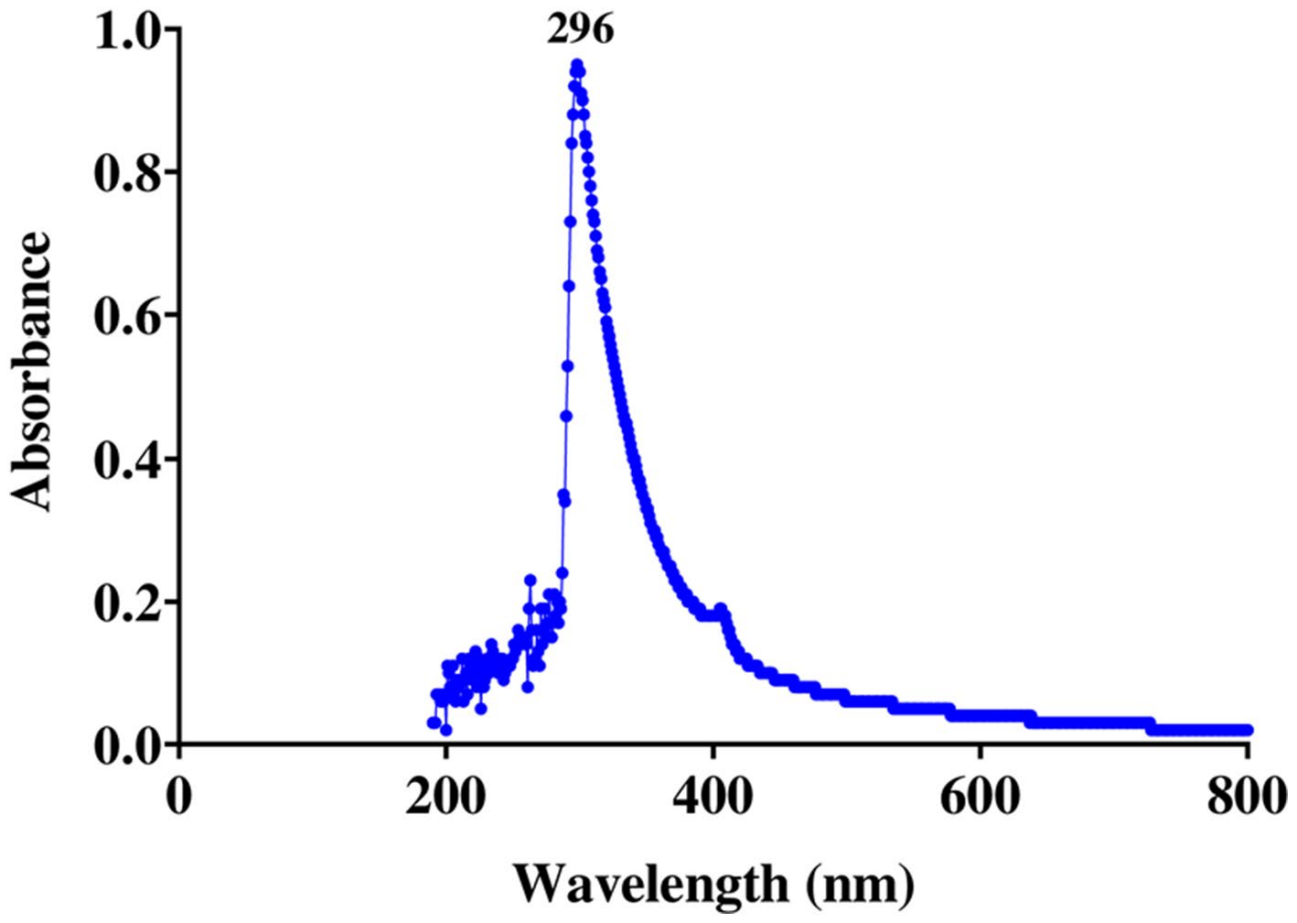
UV-visible spectrum of the crude ethyl acetate extract of *Lentzea* sp. isolate E25-2.

Spectral examination of the ethyl acetate extract from isolate E25-2 indicated the absence of polyene molecules (Figure 8). Polyene molecules are generally defined by their tendency to exhibit three distinct absorption maxima in the UV-visible range, located between 291 and 405 nm.

### 3.8 Evaluating toxicity of E25-2 isolate using *in vitro* method hemolysis

**Figure 9:**
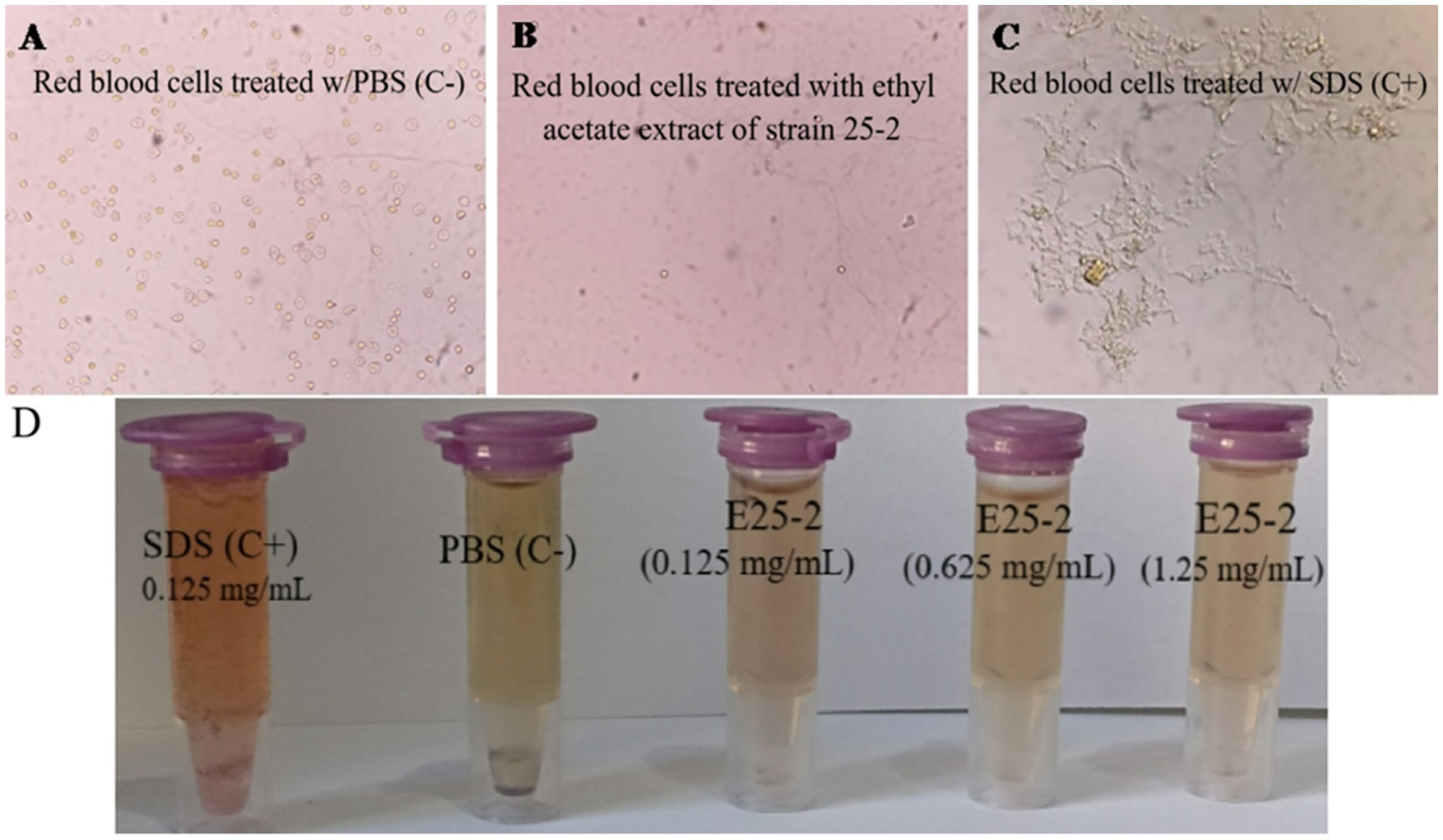
Evaluation of the PBS, ethyl acetate extract E25-2, and SDS effect on red blood cells: a comparative microscopic study. **(A)** Microscopic image showing the effect of PBS (negative control), **(B)** ethyl acetate extract, and **(C)** SDS (positive control) on red blood cells treated in different concentrations. **(D)** The ethyl acetate extract toxicity evaluation of strain E25-2 using the hemolysis test on human red blood cells.

As shown in Figure 9, no visible hemolysis was observed at different concentrations (0.125 to 1.25 mg/mL) of the ethyl acetate extract from isolate E25-2. Additionally, quantitative determination of hemoglobin by UV-vis spectroscopy also demonstrated negligible hemolytic activity of the ethyl acetate extract from isolate E25-2.

### 3.9 Gas chromatography-mass spectrometry analysis

**Figure 10:**
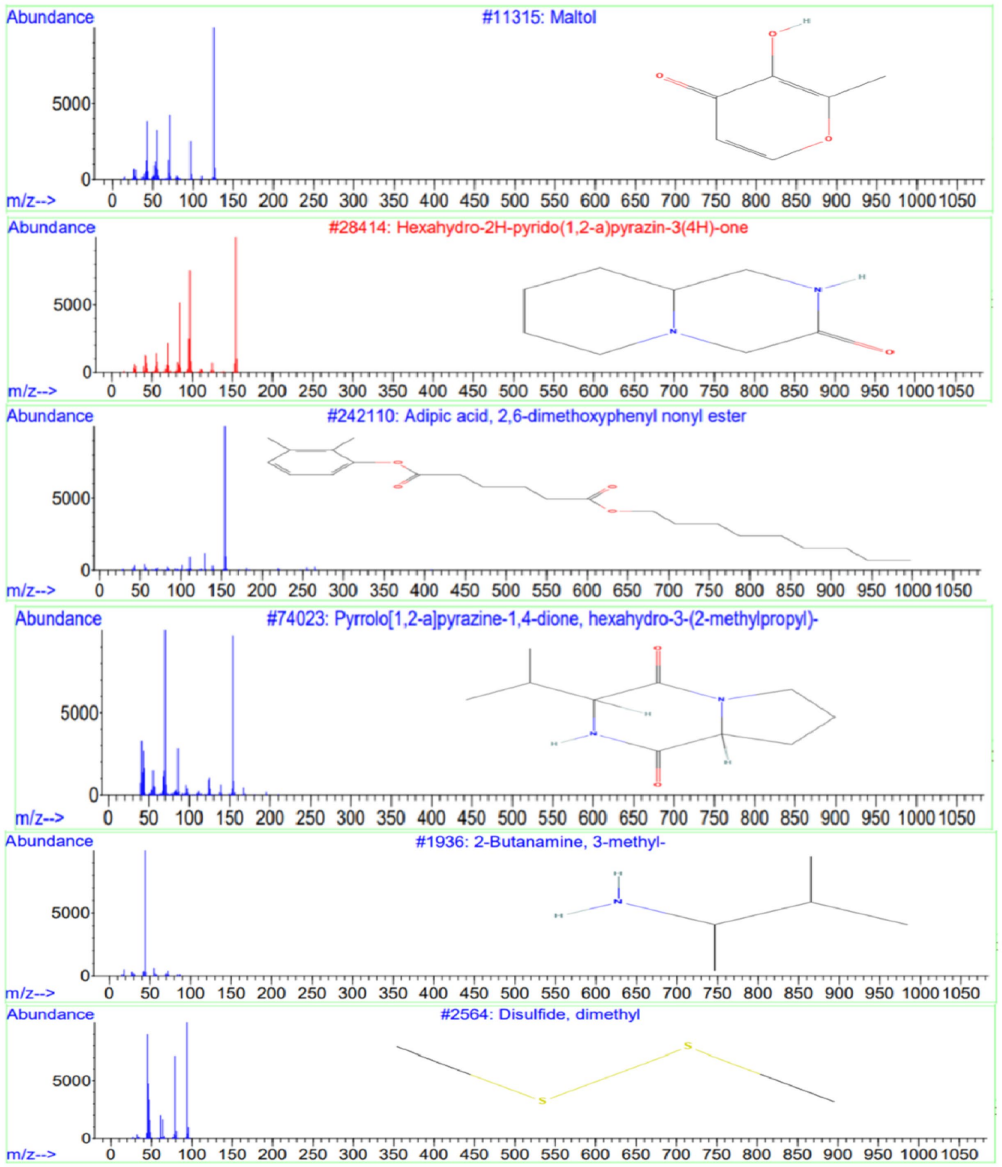
Chemical structures and mass spectra of 4 important secondary metabolites present in ethyl acetate extract of strain E25-2 analyzed by GC-MS.

GC-MS analysis of the ethyl acetate extract from isolate E25-2 indicated the presence of amines, hydroxyl groups, pyrido-pyrazinone rings, esters, and pyrrolopyrazines. These chemical compounds were identified by comparing their mass spectra with the NIST library database. Detailed information regarding the chemical compounds, including their retention time, molecular weight, and molecular formula, is provided in Table 4. Moreover, their respective chemical structures are illustrated in Figure 10.

**Table 4:** Chemical compounds identified by GC-MS in E25-2 extract.

T (Time)	Area (%)	M.W. (g/mol)	Molecular formula	Compound name	Reported bioactivity
9.19	2.73	94.20	C_2_H_6_S_2_	Disulfide, dimethyl	Antioxidant, antifungal, analgesic effect (Morales-López et al., 2017; Pozsgai et al., 2017; Tyagi et al., 2020)
10.43	0.38	87.16	C_5_H_13_N	2-Butanamine, 3-methyl-	Antimicrobial activity (Hameed et al., 2018)
10.62	0.60	126.11	C_6_H_6_O_3_	Maltol	Antioxidant, anti-inflammatory, and antitumor (Han Y. et al., 2015)
24.88	0.86	154.21	C_8_H_14_N_2_O	Hexahydro-2H-pyrido (1,2)pyrazin- 3(4H)-one	Antimycobacterial, antibacterial, antifungal, antidiabetic, diuretic, anticancer, antiviral, hypnotic, and analgesic (Dolezal and Zitko, 2015)
26.73	0.95	376.52	C_23_H_36_O_4_	Adipic acid, 2,6-dimethylphenyl nonyl ester	Not yet reported
27.10	1.39	210.27	C_11_H_18_N_2_O_2_	3-isobutyl-hexahydro-pyrrolo[1,2-a] pyrazine-1,4-dione	Antibacterial (Ser et al., 2015; Sanjenbam and Kannabiran, 2016; Raharja et al., 2019), fungicidal activity (Basavanna et al., 2021). Antifungal, antioxidant, and insecticidal (Ho, 2012)

RT, retention time; M.W., molecular weight.

## 4 Discussion

The isolate named E25-2 was classified as belonging to the genus *Lentzea*. Based on the results of the phylogenetic analysis, strain E25-2 has a 16S rRNA sequence similarity of 96.10% with *Lentzea flaviverrucosa* AS4.0578 NR_028763. Compared with the closest known strains, such as *Lentzea flaviverrucosa*, the sequence similarity is less than 98.7% (96.10%), suggesting the possibility that this is a new taxon according to Rossi-Tamisier et al. (2015). This result was confirmed by EzBioCloud databases, which also indicated a sequence similarity of 96.10% with the same closest taxon (Supplementary Table S4). These findings reinforce the hypothesis that strain E25-2 may represent a distinct new taxon within the genus *Lentzea*.

In terms of phenotype, isolate E25-2 has a segmented, branched, whitish-colored, non-powdery aerial mycelium and a yellow-orange vegetative mycelium. This study also allowed us to characterize isolate E25-2 in terms of physiological, biochemical and micromorphological characteristics, to enhance our understanding of the isolate. Moreover, E25-2 can assimilate all carbohydrate compounds but it is unable to utilize D-arabinose and melezitose. These phenotypic characteristics of isolate E25-2 are similar to the *Lentzea* genus, it is Gram-positive, aerobic, non-motile bacteria that form highly branched substrates and segregated aerial mycelia, and it unable to assimilate D-arabinose and melezitose (Maiti and Mandal, 2022). Consistent with the description provided by Maiti and Mandal (2022), members of the *Lentzea* genus, including isolate E25-2, demonstrate an inability to assimilate D-arabinose and melezitose. The majority of *Actinomycetes* species thrive in mesophilic conditions, displaying optimal growth temperatures ranging from 25 to 35°C, and a preferred pH level between 6.5 and 8.0 (Goodfellow et al., 2012; Barka et al., 2016). Optimum growth for isolate E25-2 is between 28 and 37°C. No growth was observed at 4°C or 46°C. These findings align closely with the outcomes reported by Singh et al. (2012).

*Lentzea* species, in particular those belonging to group 692 (Maiti and Mandal, 2022), appear to be a diverse source of genes involved in the production of secondary metabolites with pharmaceutical importance including, antimicrobial, antioxidant, anticancer, antibacterial, antimycobacterial, antifungal, antiviral, insecticidal, antiparasitic and other properties associated with natural products (Maiti and Mandal, 2022). Isolate E25-2 has demonstrated considerable antimicrobial activity against Gram-positive and Gram-negative bacteria, as well as phytopathogenic fungi and yeasts. According to the results of the antimicrobial test, *Lentzea* sp. E25-2 demonstrated antimicrobial activity against MDR pathogenic bacteria, as well as against phytopathogenic fungi and yeasts. According to the literature, the *Streptomyces* genus is recognized for its ability to naturally produce secondary metabolites with antimicrobial properties, and has strong biocontrol potential, acting effectively against MDR bacteria and phytopathogenic fungi such as *Fusarium* sp. (de Lima Procópio et al., 2012; Rammali et al., 2022). However, the antimicrobial activity of the *Lentzea* genus has rarely been reported in the literature. In our study, the *Lentzea* sp. E25-2 isolate could be considered an important source for the exploration of antimicrobial compounds, opening up promising prospects for potential applications in this field.

Reactive oxygen species (ROS), are reactive molecules that have the potential to inflict damage on nucleic acids, proteins, carbohydrates, and lipids, contributing to various disorders such as oxidative stress, aging, cancer, and immune dysfunction (Santos-Sánchez et al., 2019). Antioxidants have the ability to slow down or inhibit free radicals reducing oxidative stress and preventing cellular damage (Rammali et al., 2022). Microorganisms such as Actinomycetes efficiently produce a variety of natural bioactive molecules, including antioxidants (Chandra et al., 2020). A more recent study has shown that *Lentzea* genomes contain at least 53 genes for mitigating oxidative stress (Maiti and Mandal, 2022). Furthermore, existing literature suggests that relying on a solitary test is insufficient for a comprehensive evaluation of the antioxidant activity present in extracts (Tirzitis and Bartosz, 2010; Gupta, 2015; Law et al., 2019). Therefore, in order to obtain a more in-depth assessment, three *in vitro* tests including DPPH, ABTS and FRAP, were carried out. These three complementary methods provide a more complete picture of the antioxidant capacity of ethyl acetate extract from isolate E25-2. The ethyl acetate extract derived from *Lentzea* sp. E25-2 demonstrated higher antioxidant properties, suggesting the presence of bioactive compounds with diverse mechanisms of antioxidant activity. These findings imply that the *Lentzea* strain might possess the capability to produce one or more antioxidants, potentially valuable in preventing oxidative stress. To our knowledge, no strain of the *Lentzea* genus has been tested against DPPH, ABTS and FRAP free radicals to explore its antioxidant capacity. Phenolic compounds, characterized by an aromatic ring with hydroxyl groups, are recognized for their antioxidant properties (Balasundram et al., 2006). Furthermore, these substances exhibit additional advantageous bioactivities (Balasundram et al., 2006; Rammali et al., 2022). The statistical analysis conducted in this investigation substantiates this proposition by revealing a significant correlation (*p* < 0.0001) between the antioxidant activity of the ethyl acetate extract, assessed through DPPH, ABTS, and FRAP tests, and the concentrations of total phenolics and flavonoids.

Due to the structural similarity between human cholesterol and ergosterol, predominant in fungal cells, polyene antifungal molecules interact with cholesterol, disrupting fungal cell membranes and leading to cell death. This interaction with human cholesterol is associated with potential toxicity. As a result, researchers often avoid incorporating these molecules into research programs for new bioactive substances (Yilma et al., 2007). In this context, the strain studied, *Lentzea* sp. E25-2, is particularly interesting, as it is devoid of toxic polyene molecules, which are detected by three specific absorption maxima in the UV-visible range between 291 and 405 nm (Aouiche et al., 2012). This characteristic suggests that this strain could be promising for the discovery of new bioactive molecules, particularly in the antifungal field.

A hemolytic assay was conducted to assess the impact of bioactive compounds present in the ethyl acetate extract of isolate E25-2 on red blood cell membrane disruption. Several studies have demonstrated that the *in vitro* hemolysis test correlates well with *in vivo* toxicity via the hemolytic effect (Lu et al., 2009). Hemoglobin is a protein found in red blood cells, playing a crucial role in transporting oxygen from the lungs to the tissues of the body. However, the hemoglobin released by hemolysis can break down into by-products, some of which can contribute to vasoactive reactions, resulting in damage to various vital organs such as the liver, kidneys and heart (Buehler and D’Agnillo, 2010). Consequently, the extract showed no membranolytic activity on the erythrocyte membrane at all the tested concentrations. According to Saurav and Kannabiran (2012), evaluating membrane stability during exposure to new drugs is crucial, and erythrocytes serve as a suitable model for studying this stability. The impact of various bioactive compounds on the mechanical stability of the erythrocyte membrane serves as a reliable indicator of overall membrane stability.

GC-MS analysis was conducted to identify the bioactive compounds present in the ethyl acetate extract of isolate E25-2. The compounds identified in this study include phenolic compounds, characterized by an aromatic ring carrying one or more hydroxyl (OH) groups. These compounds are renowned for their well-documented antioxidant properties. The phenolic compound detected in the ethyl acetate extract is maltol, whose structure includes a phenol group, represented by the aromatic ring. This compound has been shown to have antioxidant, anti-inflammatory and anti-tumor activity. The compounds identified encompass phenolic compounds characterized by an aromatic ring containing one or more hydroxyl (OH) groups, renowned for their antioxidant properties (Balasundram et al., 2006). Within the ethyl acetate extract, maltol, a phenolic compound with a structure featuring a phenol group within the aromatic ring, was detected. Previous study has demonstrated that maltol exhibits antioxidant, anti-inflammatory, and anti-tumor activities (Han H. et al., 2015).

Phenolic compounds could be a natural source of physiological properties, such as antioxidant effects and other bioactivities (Balasundram et al., 2006). Heterocyclic compounds were also identified in the ethyl acetate extract, including pyrazines and pyrrolopyrazines. Pyrazines, heterocyclic compounds commonly found in nature, are generally produced by microorganisms (Schulz and Dickschat, 2007). Some pyrazines have been associated with beneficial activities such as antioxidants, anticancer and antimicrobial properties (Ser et al., 2016). The compound Hexahydro-2H-pyrido (1,2-a) pyrazin-3(4H)-one has been identified in the ethyl acetate extract of isolate E25-2 and has been reported in the literature to exhibit various properties, such as antifungal, diuretic, antidiabetic, anticancer, antibacterial, antiviral, hypnotic, analgesic activities, and antimycobacterial (Dolezal and Zitko, 2015).

Pyrrolopyrazines are known for their diverse range of bioactivities, which include antioxidant, antitumor, antibacterial, antifungal, and anti-angiogenic properties (Ser et al., 2015). Additionally, the compound “3-isobutyl-hexahydro-pyrrolo[1,2-a]pyrazine-1,4-dione-” was detected in the ethyl acetate extract and has been reported in the literature to exhibit various properties, such as antibacterial (Ser et al., 2015; Sanjenbam and Kannabiran, 2016; Raharja et al., 2019), fungicidal (Basavanna et al., 2021), antioxidant and insecticidal (Ho, 2012). Furthermore, the compound disulfide, dimethyl detected in the ethyl acetate extract has previously been reported to exhibit various biological activities, including antioxidant, antifungal, and analgesic effects (Morales-López et al., 2017; Pozsgai et al., 2017; Tyagi et al., 2020). Similarly, the compound 2-Butanamine, 3-methyl- detected in the extract was previously reported to possess antimicrobial activities (Hameed et al., 2018).

Overall, the chemical compounds identified in the current study are well known for their antimicrobial and antioxidant activities, indicating that these components could be responsible for the antioxidant and antimicrobial activity of the ethyl acetate extract of *Lentzea* sp. E25-2. Consequently, this study provides further evidence of the potential of *Lentzea* sp. E25-2 derived from Moroccan forest soil as a promising source of antimicrobial and antioxidant agents. However, further research is needed to precisely identify the individual compound or combination of compounds responsible for the observed activities.

## 5 Conclusion

In conclusion, the isolate E25-2, derived from Moroccan forest soil, was classified as belonging to the genus *Lentzea* based on 16S rRNA sequencing and phylogenetic analysis. Notably, E25-2 exhibited substantial antimicrobial activity against a range of Gram-positive and Gram-negative bacteria, phytopathogenic fungi, and yeast, with ethyl acetate identified as the most effective solvent for extracting antimicrobial agents. The ethyl acetate extract of E25-2 showed significant antioxidant properties, as evidenced by DPPH, ABTS, and FRAP assays, which correlated strongly with its phenolic and flavonoid contents. The ethyl acetate extract analysis by GCMS identified various bioactive compounds, including pyrazines, phenolics, and pyrrolopyrazines, known for their antimicrobial and antioxidant activities. Importantly, the extract was found to be non-toxic, as indicated by the absence of polyene molecules and negligible hemolytic activity. Our results suggest that *Lentzea* sp. E25-2 is a promising source of novel bioactive compounds with prospective pharmaceutical uses, particularly as antimicrobial and antioxidant agents. This highlights the soil-derived microorganisms’ value in the new natural products discovery. Further research is needed to isolate and characterize the specific compounds responsible for the bioactivities observed in *Lentzea* sp. E25-2, which could lead to new therapeutic agents’ development.

## Data availability statement

The original contributions presented in the study are included in the article/Supplementary material, further inquiries can be directed to the corresponding author.

## Author contributions

SR: Writing – original draft, Writing – review & editing. AC: Writing – original draft, Writing – review & editing. MA: Writing – original draft, Writing – review & editing. AR: Writing – original draft, Writing – review & editing. FK: Writing – original draft, Writing – review & editing. KD: Writing – original draft, Writing – review & editing. AK: Writing – original draft, Writing – review & editing. LR: Writing – original draft, Writing – review & editing. BN: Writing – original draft, Writing – review & editing. AP: Writing – original draft, Writing – review & editing. BB: Writing – original draft, Writing – review & editing.
