# Supplementary material for: Exploring the antimicrobial and antioxidant properties of Lentzea flaviverrucosa strain E25-2 isolated from Moroccan forest soil
Source: Front Microbiol. 2024 Jul 22;15:1429035. doi: 10.3389/fmicb.2024.1429035 (PMC11298423; doi:10.3389/fmicb.2024.1429035)
Supplement: Supplementary file 1 [file Table_1.DOCX]

Supplementary Material

# General methods

## Recommended media for *Actinobacteria* isolation

M2: 10 g of starch, 0.3 g of casein, 2 g of KNO_3,_2 g of NaCl, 0.05 g of MgSO_4_,7H_2_O, 2 g of K_2_HPO_4,_ 0.02 g of CaCO_3_, 0.01 g of FeSO_4_, 7H_2_O, 1 g of glucose, 15 g of agar, 1L distilled water, and pH adjusted at 7. 2 (Bouaziz et al., 2016).

GA: 10 g of Glucose, 0.5 g of asparagine, 0.5 g of K_2_HPO_4_, 15 g of agar, 1L distilled water, and pH adjusted at 6 (Bouaziz et al., 2016).

GLM: 10 g of glucose, 3 g of yeast extract, 3 g of malate extract, 5 g of tryptone, 15 g of agar, 1L distilled water, and pH adjusted at 7.2 (Thakur et al., 2007; Das et al., 2018).

Bennett : 10 g of glucose, 2 g of yeast extract, 1 g of meat extract, 2 g of tryptone, 15 g of agar, 1L distilled water, and pH adjusted at 7.2 (Lee et al., 2011).

## Recommended media for testing antimicrobial activity (Badji et al., 2005)

ISP1: 3 g of yeast extract, 5 g of tryptone, 16 g of agar, 1L of distilled water, and pH adjusted at 7.15.

ISP2: 4 g of yeast extract, 4 g of glucose, 10 g of malate extract, 16 g of agar, 1L of distilled water, and pH adjusted at 6.51.

GYEA: 10 g of yeast extract, 10 g of glucose, 16 g of agar, 1L of distilled water, and pH adjusted at 6.96.

Bennett: 10 g of glucose, 2 g of yeast extract, 1 g of meat extract, 2 g of tryptone, 15 g of agar, 1L distilled water, and pH adjusted at 7.2.

## Recommended medium for testing NaCl tolerance

YEA medium: 3 g of yeast extract, 5 g of peptone, 15 g of agar, 1L of distilled water, and pH adjusted at 7.2.

## Recommended medium for the production of melanoid pigment

ISP9: 2.64 g of (NH_2_) SO_4_, 2.38 g of KH_2_PO_4_, 5.65 g of K_2_HPO_4_, 1 g of MgSO_4_, 7H_2_O, 20 g of agar, and 1L of distilled water.

100 mL salt solution: 4 g of CuSO_4_, 5H_2_O, 1.1 g of FeSO_4_, 7H_2_O, 7.9 g of MnCl_2_, 4H_2_O, 1.5 g of ZnSO_4_, 7H_2_O.

GYEA: 10 g of yeast extract, 10 g of glucose, 16 g of agar, 1L of distilled water, and pH adjusted at 6.96.

## Other media used in this study

PDA medium: 4 g of potato extract 20 g of glucose, 15 g of agar, 1L distilled water, and pH adjusted at 7.5.

Muller Hinton Agar medium: Ready to use

CLED medium: Ready to use.

# Supplementary Figures and Tables

## Supplementary Figures


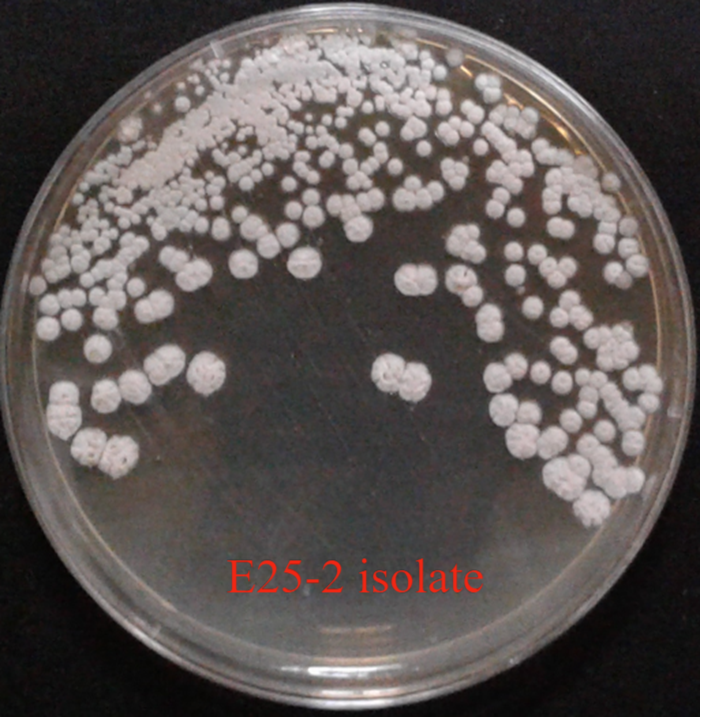


**Supplementary Figure 1.** Morphological aspect of *Lentzea* sp. E25-2.


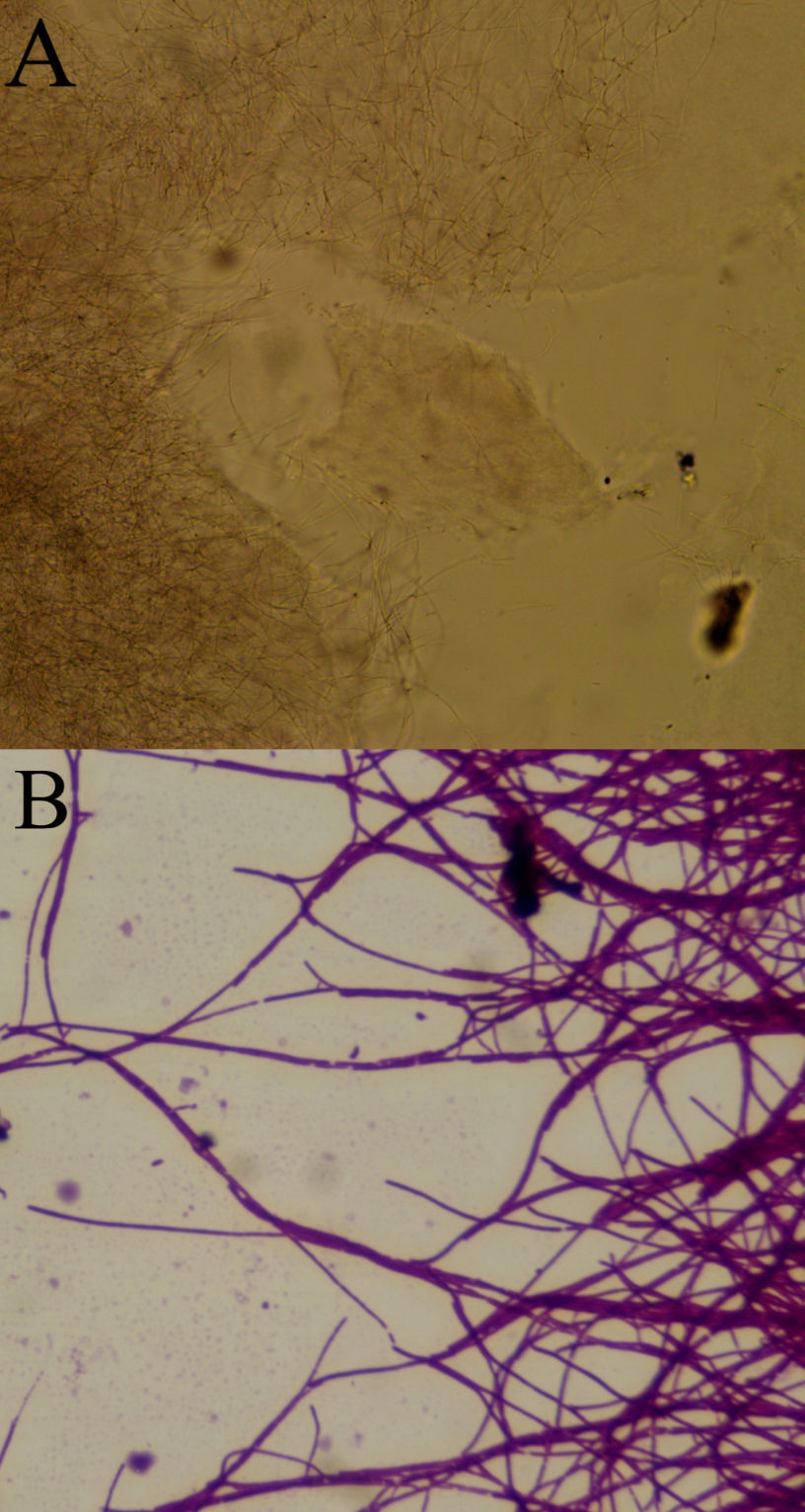


**Supplementary Figure 2.** Optical microscope of *Lentzea* sp. E25-2 (A: fresh, B: after Gram staining).

## Supplementary Tables

**Supplementary Table 1**. Molecular identification of strain E25-2 based on 16S rRNA gene sequencing (NCBI’s nucleotide BLAST program).

| **Isolate Name** | **Query**  **Length**  **(bp)** | **Nearest known species^a^** | **Identification^b^**  **(Scientific Name)** | **Percent Identity**  **(%)** | **E value** | **Query Coverage**  **(%)** | **Total**  **score** | **Max score** | **Accession**  **length** | **NCBI GenBank accession number** |
| --- | --- | --- | --- | --- | --- | --- | --- | --- | --- | --- |
| E25-2 | 1103 | *Lentzea flaviverrucosa* AS4.0578^T^ (NR_028763.1) | *Lentzea flaviverrucosa* | 96.10 | 0.0 | 97 | 1735 | 1735 | 1426 | OR865862 |

^a^NCBI’s nucleotide BLAST program was used to find the closest match against the non-redundant reference RNA sequence database (refseqrna). The strain number, strain type (T), and GenBank accession number are all listed after the species name.

^b^Isolates with a greater percentage of identity than 96.78% and only one closest match for the same identity characteristics are classified up to the species level. To assess the reliability of a phylogenetic tree, MEGA provides the Bootstrap test. This test uses the bootstrap re-sampling strategy, so you need to enter the number of replicates. For a given data set, applicable tests and the phylogeny inference method are enabled. Neighbor joining has an additional test (Interior Branch), which requires the same input as bootstrap.

| Isolate  name | Identification  (scientific name) | NCBI GenBank  accession number | Colony  aspect | Colony diameter (mm) | Surface (aerial mycelium) | Reverse (substrate mycelium) | Pigmentation of the medium | Gram  staining |
| --- | --- | --- | --- | --- | --- | --- | --- | --- |
| E25-2 | *Lentzea flaviverrucosa* | OR865862 | Starred | 1-6 | Whitish, not powdery | Yellowish, orange | Not present | Positive filaments |

**Supplementary Table 2.** Morphological and micromorphological characteristics of *Actinobacteria* isolate E25-2.

| Test strains MDR | Resistance to | Sensitivity to | Intermediate to | Families |
| --- | --- | --- | --- | --- |
| *Escherichia coli* 16D1150 | P, AMP, AML, AMC, CXM, CTX, FD, SXT, CIP, OFX, VA | CRO | CN, F | Penicillins, cephalosporins, steroids, quinolones, sulfonamides and Glycopeptides |
| *Proteus vulgaris* 16C1737 | P, AMP, AML, AMC, CXM, CTX, SXT, FD, F | CN, CRO, OFX, | CIP, AK | Penicillins, cephalosporins, sulfonamides and steroids |
| *Neisseria gonrrhoae* 16D1170 | P, AMP, AML, NA, FMQ, OFX, TE, SXT, FD, | AMC, CRO, AN, CN, CRO, E, F | CIP, | Penicillins, quinolones, tetracyclines, steroidal and sulfonamides |
| *Staphylococcus aureus*18K1052 | P, AMP, AMX, AMC, CRO, CIP, NA, FMQ, OFX. | KF, CXM, AN, GN, E, SXT, FD, IPM, F | - | Penicillins, cephalosporins and quinolones |
| *Enterococcus faecalis* 18K1386 | P, AMP, KF, CXM, CRO, AN, GN, CIP, NA, FMQ, OFX, E, FD | AMX, AMC,  SXT, F | IPM | Penicillins, cephalosporins, aminosides, quinolones, erythromycin and steroids |

**Supplementary Table 3.** Resistance profile of multi-drug resistant (MDR) clinical bacteria.

AMC : Amoxicillin + Ac.clavulanic; P: penicillin G; AMP: Ampicillin; KF: Cefalotin; CRO: Ceftriaxone; AN: Amikacin; CN: Gentamicin; CIP: Ciprofloxacin; NA: Alidixicacid; FMQ: Flumequie; OFX: Ofolxacin; E: Erythromycin; TE: Tetracycline SXT: Trimethoprim+Sulfamide; FD: Fusidicacid; F: Nitrofurantne; CXM: Cefuroxin; VA: Vaomycin; CTX: Cefotaxime. IPM:Imipenem; CAZ: Ceftazidim; TM: Tobramycin; FOX: Cefoxitin; TIC: Ticarcillin.

**Supplementary Table 4.** Molecular identification of strain E25-2 based on 16S rRNA gene sequencing (EzBioCloud’s identification).

| **Name** | **Top-hit**  **taxon** | **Top-hit strain** | **Similarity (%)** | **Top-hit taxonomy** | **Completeness**  **(%)** |
| --- | --- | --- | --- | --- | --- |
| E25-2 | *Lentzea flaviverrucosa* | AS4.0578 | 96.10 | Bacteria, *Actinobacteria*, *Actinomycetia*, *Pseudonocardiales*, *Pseudonocardiaceae*, *Lentzea* | 75.70 |

# References

Badji, B., Riba, A., Mathieu, F., Lebrihi, A., and Sabaou, N. (2005). Activité antifongique d’une souche d’Actinomadura d’origine saharienne sur divers champignons pathogènes et toxinogènes. *J. Mycol. Med.* 15, 211–219.

Bouaziz, S., Messis, A., Bettache, A., El Hadj, M. D. O., and Benallaoua, E. S. (2016). Antifungal activity of Streptomyces sp. 14 strain isolated from Ouargla (Southeast of Algeria): Identification, production and characterization of the active substance. *Int. J. Biosci* 9, 45–56.

Das, R., Romi, W., Das, R., Sharma, H. K., and Thakur, D. (2018). Antimicrobial potentiality of actinobacteria isolated from two microbiologically unexplored forest ecosystems of Northeast India. *BMC Microbiol.* 18, 1–16.

Lee, E. J., Hwang, K. Y., Lee, H.-S., and Chung, N. (2011). Characterization of a new Streptomyces sp. A1022 as a potential biocontrol agent. *J. Korean Soc. Appl. Biol. Chem.* 54, 488–493.

Thakur, D., Yadav, A., Gogoi, B. K., and Bora, T. C. (2007). Isolation and screening of Streptomyces in soil of protected forest areas from the states of Assam and Tripura, India, for antimicrobial metabolites. *J. Mycol. Med.* 17, 242–249.
